# Supplementary material for: Patterns of Intron Gain and Loss in Fungi
Source: PLoS Biol. 2004 Nov 30;2(12):e422. doi: 10.1371/journal.pbio.0020422 (PMC532390; doi:10.1371/journal.pbio.0020422)
Supplement: Table S1 — Also available at http://genes.mit.edu/NielsenEtAl/. (4.3 MB ZIP). [file pbio.0020422.st001.zip › NielsenEtAl/html/757.html]

AN2508.1.NCU04636.1.MG00767.1.FG10049.1


```
 CLUSTAL W (1.82) Multiple Sequence Alignments - Introns Inserted


Sequence 1: MG00767.1	513 aa
Sequence 2: FG10049.1	501 aa
Sequence 3: NCU04636.1	505 aa
Sequence 4: AN2508.1	507 aa
Alignment Length: 521 aa
Number Identitical Residues: 347 aa
Alignment Score (without introns) 14470


MG00767.1 	-MSTIAASALRQASRLACRPSASTASWQAIRGGARASGAASLRKSAPARTYVSETKRDNA
NCU04636.1	-MSTIAPSVLRQAS-----RLAAAAPLRSARVLSRATTAAAVKTASASRSYVTETKRNNA
FG10049.1 	MSSRIASCALRSAG----RVSLSSGSRLTVAP-LRMARTAAVARLTSQRGYVTETKRDNA
AN2508.1  	-MSSVAPFALKQAS-------RAYARRLTAAQHALPWRALATP---SYRTFVSETKAGNA
          	  * :*. .*:.*.        : .   :         : :     . * :*:*** .**

MG00767.1 	QVAEANIETAIHLDKKDFERIG-------LAMESQGGSDVAVSPMAE1ILKGAAVMEEGQ
NCU04636.1	DVQ---AEHAIKLDHREMEKQG-------LTISAQNGSSQHVSPMAD1VLSNATVMDEGQ
FG10049.1 	QV-----ETAIKLDKTAFADIP-------PPGTP---SNAKVSPMAE1VLKSAAVMEEGQ
AN2508.1  	QVS---VDTAIKQEQKDFLKQTGVQPQNAELPASGITGDTAMSPAAG~ILKQATIMDQGT
          	:*     : **: ::  :    . ....:    .   ..  :** *  :*. *::*::* 

MG00767.1 	RPIYLDMQATTPVDPRVLDAMLPLYVGVYGNPHSRTHAYGWESEKAVEDARAHVASLIGA
NCU04636.1	RPIYLDMQATTPIDPRVLDAMMPYFTNVYGNPHSRTHAYGWETDKAVEEARKHIADLIGA
FG10049.1 	RPIYLDMQATTPVDPRVLDAMMPFYVGVYGNPHSRTHAYGWESEKAVEDAREHVAKLIGA
AN2508.1  	RPIYLDAQATTPTDPRVLDAMLPYLTGIYGNPHSRTHAYGWESEKAVEQARGYVANLIGA
          	****** ***** ********:*  ..:**************::****:** ::*.****

MG00767.1 	DPKEIIFTSGATESNNMSIKGVARFFGRSGKKKHIITTQTEHKCVLDSCRHLQDEGFEVT
NCU04636.1	DPKEIIFTSGATESNNMSIKGVARFFGRSGKKKHIITSQTEHKCVLDSCRHLQDEGFEVT
FG10049.1 	DSKEIIFTSGATESNNMSIKGVARFFGRSGKKKHIITTQTEHKCVLDSCRHLQDEGFEVT
AN2508.1  	DAKEIIFTSGATESNNMSIKGVARFFGRSGKKKHIITSQTEHKCVLDSCRHLQDEGFDVT
          	*.***********************************:*******************:**

MG00767.1 	YLPVQNSGLVDLKELEAAMRPETALVSIMTVNNEIGVIQPVEEIGKMCRERKIFFHTDAA
NCU04636.1	YLPVKSSGLIDMAELEAAIRPDTAIVSIMAVNNEIGVIQPLEEIGKLCRSKKIFFHTDAA
FG10049.1 	YLPVQNDGLIKMEDLEAAIRPETALVSIMTVNNEIGVIQPIEQIGKLCRSKKIFFHTDAA
AN2508.1  	YLPVQSNGLIKLEDLEAAMRPDTALVSIMAVNNEIGVVQPLEEIGKLCRSKKIFFHTDAA
          	****:..**:.: :****:**:**:****:*******:**:*:***:**.:*********

MG00767.1 	QAVGKIPMDVNAMNIDLMSISGHKIYGPKGIGACYVRRRPRVRLDPIISGGGQERGLRSG
NCU04636.1	QAVGKIPVDVNAMNIDLMSISSHKIYGPKGIGACYVRRRPRVRLDPIISGGGQERGLRSG
FG10049.1 	QAVGKIPLDVNAMNIDLMSISSHKIYGPKGIGACYVRRRPRVRLDPLITGGGQERGLRSG
AN2508.1  	QAVGKIPIDVNKMNIDLLSVSSHKIYGPKGIGACYVRRRPRVRLEPIISGGGQERGLRSG
          	*******:*** *****:*:*.**********************:*:*:***********

MG00767.1 	TLAPPLIVGFGEACRIAKQEME0YDSKRVKYLSDRLLKGLLSMEHTSQNGHPDHFYPGCV
NCU04636.1	TLAPPLVVGFGEACRIAKEEMP0YDSKRIKHLSDRLLNGLLSMEHTSQNGDPNHFYPGCV
FG10049.1 	TLAPSLIAGFGEACRIAKEEMP0YDTNRIKYLSDRLLNGLLSMEHTTQNGAPESFYPGCV
AN2508.1  	TLAPHLVVGFGEACRVAAQDME0YDSKYISRLSKRLTDGLLAMEHTSLNGDPERRYPGCV
          	**** *:.*******:* ::*  **:: :. **.** .***:****: ** *:  *****

MG00767.1 	NVSFAYVEGESLLMALKDIALSSGSACTSASLEPSYVLRALGNSDESAHSSIRFGIGRFT
NCU04636.1	NVSFAYVEGESLLMALKDIALSSGSACTSASLEPSYVLRALGNSDESAHSSIRFGIGRFT
FG10049.1 	NVSFAYVEGESLLMALKDIALSSGSACTSASLEPSYVLRALGNSDESAHSSIRFGIGRFT
AN2508.1  	NVSFAYVEGESLLMALKDIALSSGSACTSASLEPSYVLRALGSSDESAHSSIRFGIGRFT
          	******************************************.*****************

MG00767.1 	TEREIDYVLKAVQERVSFLRELSPLWELVQEGVDLNTIQWSQH
NCU04636.1	TEQEIDYVLKAVTERVGFLRELSPLWELVQEGIDLNTIQWSQH
FG10049.1 	TEAEIDYVLKAVQERVSFLRELSPLWELVQEGIDLDTIQWSQH
AN2508.1  	TEAEIDYVLKAVQERVHFLRELSPLWELVQEGIDLNTIEWSGH
          	** ********* *** ***************:**:**:** *
```
